# ATG9B-4 accelerates the proliferation and migration of liver cancer cells in an ARNTL–CDK5 pathway-dependent manner: A case–control study

**DOI:** 10.1097/MD.0000000000042227

**Published:** 2025-04-18

**Authors:** Ming Li, Jiefu Tang, Wenxiong Zhu, Changshen Cheng, Lili Guo, Pinyue Liu, Zhongcheng Mo

**Affiliations:** a School of Basic Medical Sciences, The First Affiliated Hospital, Hunan University of Medicine, Huaihua, Hunan, China; b Department of Orthopaedics, Dongguan People’s Hospital, Dongguan, Guangdong, China; c Department of Hepatology, Guidong People’s Hospital of Guangxi Zhuang Autonomous Region, Affiliated Guidong People’s Hospital of Guilin Medical University, Wuzhou, Guangxi, China; d Department of Orthopaedics, Sanming First Hospital Affiliated to Fujian Medical University, Sanming, Fujian, China; e Department of Histology and Embryology, Guangxi Key Laboratory of Diabetic Systems Medicine, Guilin Medical University, Guilin, Guangxi, China.

**Keywords:** ARNTL, ATG9B-4, liver cancer, migration and proliferation

## Abstract

Lnc ATG9B-4 aggravated the progression of liver cancer by up-regulating cyclin-dependent-kinase 5 (CDK5). It could be inferred that ATG9B-4 indirectly regulates the expression of CDK5 via lncRNA-mediated negative regulation of target genes. Therefore, the specific molecular mechanism by which ATG9B-4 regulates the malignant characteristics of liver cancer cells still needs further study. The differentially expressed genes were identified by mRNA sequencing in liver cancer cells transfected with or without ATG9B-4. Liver cancer cells were transfected with ATG9B-4, ARNTL, or si-CDK5. The expression of aryl basic helix–loop–helix ARNT like 1 (BMAL1, also known as ARNTL), CDK5, and ATG9B-4 was analyzed by real-time quantitative PCR and western blotting. The proliferation and invasion of the transfected cells were respectively analyzed by cell counting kit-8 and wound healing assays, respectively. The ARNTL expression was down-regulated in the liver cancer tissues and liver cancer cells transfected with ATG9B-4. Low ARNTL expression indicated poor overall survival in patients with liver cancer. The optical density of cells transfected with ATG9B-4 and ARNTL was significantly lower than that of cells transfected with ATG9B-4. The wound areas of cells transfected with ATG9B-4 and ARNTL were markedly wider than those of cells transfected with ATG9B-4. The expression of CDK5 was down-regulated in cells transfected with ARNTL. CDK5 knockdown partially attenuated the ATG9B-4-induced increase in proliferation and migration in liver cancer cells. ATG9B-4 deteriorated the proliferation and migration of liver cancer cells in an ARNTL–CDK5 pathway-dependent manner.

## 1. Introduction

Liver cancer is a common malignant tumor of the digestive system. It has been reported that the number of newly diagnosed liver cancer each year is 841,100, accounting for approximately 4.7% of new cancer cases, and the number of deaths due to liver cancer is 786,000, accounting for approximately 8.2% of cancer-related deaths worldwide.^[1]^ In 2022, 431,383 patients were newly diagnosed with liver cancer, making it rank fourth among cancers in terms of incidence, and 412,216 cases patients died due to liver cancer, making it rank second in terms of cancer mortality in China, and the incidence and mortality of liver cancer in men were greater than those in women.^[2]^ Liver cancer is one of the main malignancies, accounting for 75% to 85% of liver malignancies.^[3]^ Due to the insidious onset of liver cancer, most patients with liver cancer are already in the middle-advanced stages, and <30% of patients with liver cancer are suitable for radiotherapy at the time of first diagnosis.^[4]^ After surgery and chemotherapy, the survival of liver cancer patients in the early stage is prolonged; however, the survival of advanced liver cancer patients after systematic antitumor therapies is still unsatisfactory.^[4]^ Therefore, studying the underlying molecular mechanism of liver cancer development is helpful for the prevention and treatment of liver cancer.

Increasing evidence has suggested that long noncoding RNAs (lncRNAs) play an integral role in the progression of liver cancer. On the one hand, it was recognized that lncRNA abolished the inhibition of target genes caused by microRNA through sponge adsorption. The lnc COX7C-5 accelerated malignant characteristics, which could be reversed by the overexpression of miR-581 in liver cancer cells.^[5]^ Lnc PLA2G4A-4 facilitates the proliferation, invasion, and migration of liver cancer cells by sponging miR-23b-3p both in vitro and in vivo.^[6]^ On the other hand, a large number of studies have shown that lncRNAs can promote the expression of functional genes by inhibiting target genes. Lnc CTHCC promoted the progression of liver cancer by directly binding to hnRNPK through activating YAP1 transcription.^[7]^ Lnc UCID was up-regulated in liver cancer, and lnc UCID prevented the interaction between DHX9 and cyclin-dependent-kinase 6 (CDK6), which eliminated the inhibitory effect of DHX9 on CDK6 expression, resulting in the enhancement of the G1/S transition and liver cancer cell growth.^[8]^ LncZEB2-19 notably weakened the proliferation, metastasis, stemness, and lenvatinib resistance of liver cancer cells by specifically binding to TRA2A and promoting TRA2A degradation, which led to the instability of RSPH14 mRNA.^[9]^ According to the regulatory mechanism of lncRNAs, lncRNAs are usually negatively correlated with the genes directly regulated by lncRNAs.

In our previous study, we showed that the lncATG9B-4 promote the proliferation and migration of liver cancer cells and positively regulated the expression of cyclin-dependent kinase 5 (CDK5) in the liver cancer cells.^[10]^ According to the mechanism by which lncRNAs negatively regulate target genes, it could be inferred that lncATG9B-4 indirectly regulates the expression of CDK5. Therefore, the target genes regulated by lncATG9B-4 still need to be screened to further elucidate the regulatory loop by which ATG9B-4 inhibits the malignant characteristics of liver cancer cells.

## 2. Methods

### 2.1. Sample collection

Thirty liver cancer tissues and paracancerous normal tissues were collected at the The First Affiliated Hospital to Hunan University of Medicine from August 2014 to August 2018. These samples used in this study were the remaining tissues from pathological examination, and preserved by department of pathology. The expression of aryl basic helix–loop–helix ARNT like 1 (BMAL1, also known as ARNTL) and ATG9B-4 were analyzed by real-time quantitative PCR (RT-qPCR) in the liver cancer and normal tissues from patients with liver cancer. This project was adhered to the principles in the Declaration of Helsinki and approved by the Institution Ethics Committee of Hunan University of Medicine (HUM-2018-124). All participants signed the informed consent at the time of surgery in this study.

### 2.2. Cell culture and transfection

The HepG2 cells authenticated by STR profiling were purchased from ATCC and cultured in the DMEM (Gibco, USA) supplement with 10% FBS (C0234, Beyotime, China) and 1% penicillin–streptomycin (C0222, Beyotime, China). All cells were cultivated in a 37 ℃ incubator with 5% CO_2_ and saturated humidity. The hemo species ATG9B-4, ARNTL, and CDK5 cDNA were amplified by PCR with specific primers and inserted into pcDNA3.1 to generate the ATG9B-4, ARNTL, and CDK5 plasmids. The plasmids were amplified in *E coli*, then, extracted and purified using TIANpure Mini Plasmid Kit (DP104, TIANGEN, China) according to the instructions of manufacture. The siRNA control (si-NC, Sense: UCUCGUAGUCUGGACGUAGUCAUAU; Antisense: AUAUGACUACGUCCAGACUACGAGA) and siRNA interfering with CDK5 (si-CDK5, Sense: UAUGACAGAAUCCCAGCCCCA; Antisense: GGGCUGGGAUUCUGUCAUAAG) were designed and synthesized by Sangon Biotech (Shanghai, China). The HepG2 cells were transfected with plasmids and siRNAs using Lipofectamine 3000 (L3000015, Themo, USA) according to the instructions of manufacture. To ensure transfection efficiency, the HepG2 were transfected the same amount of plasmids or siRNAs. The cells transfected with pcDNA3.1 and si-NC were used as negative controls for the overexpression and interference experiments, respectively.

### 2.3. RT-qPCR and mRNA sequencing

The total RNAs of 30 patients with liver cancer and transfected HepG2 cells were extracted using RNA Easy Fast Tissue/Cell Kit (DP451, TIANGEN, China) according to the instructions of manufacture. The expression of ATG9B-4, ARNTL, and CDK5 were determined using FastKing One Step RT-qPCR Kit (FP313, TIANGEN, China) on CFX Connect Real-Time System (BIO-RAD, USA) with following parameters: pre-denaturation at 95 ℃ for 3 minutes; 40 cycles at 95 ℃ for 15 seconds, 60 ℃ for 30 seconds. The expression of genes were normalized by 18S and calculated with 2^-∆∆Ct^ method.^[11]^ All specific primers including PC and RT-qPCR were listed in Table 1. Meanwhile, the mRNAs of liver cancer cells transfected with or without ATG9B-4 were sequenced by GENECHEM (Shanghai, China) to identify differentially expressed genes.

**Table 1:** Primers for PCR and RT-qPCR in this study.

Purpose	Names	Sequences (5’–3’)
PCR	ATG9B-4 Forward Primer	ATCGGGATTCCAGGCAGATCACCCGAGG
	ATG9B-4 Reverse Primer	ATCGTCTAGAGACACTCCAGTCGCCAGC
	ARNTL Forward Primer	ATCGGGATTCCATGGCAGACCAGAGAATG
	ARNTL Reverse Primer	ATCGTCTAGATTACAGCGGCCATGGCAA
	CDK5 Forward Primer	ATCGGGATTCCATGCAGAAATACGAGAAA
	CDK5 Reverse Primer	ATCGTCTAGACTAGGGCGGACAGAAGTC
RT-qPCR	ATG9B-4 Forward Primer	ATTGGCTCTTTATCCCTGCTGA
	ATG9B-4 Reverse Primer	AGTGGTGCTTCCAGGATTCAA
	ARNTL Forward Primer	TCCCCTCTACCTGCTCAAAGA
	ARNTL Reverse Primer	TCGTTGTCTTCATCCAGCCC
	CDK5 Forward Primer	CTCTTTGGGGCCAAGCTGTA
	CDK5 Reverse Primer	CCTCCCTGTGGCATTGAGTT
	18S Forward Primer	AGAAACGGCTACCACATCCA
	18S Reverse Primer	CACCAGACTTGCCCTCCA

The bases highlighted with yellow, green and red are the protective bases, restriction enzyme sequences and target genes sequences, respectively.

### 2.4. Western blotting assay

The HepG2 cells transfected with or without ATG9B-4 were collected and ultrasonically lysed in precooled Cell lysis buffer (P0013, Beyotime, China) with 1 mM PMSF (P0100, Solarbio, China) on ice for 30 seconds. The supernatant of cell lysates was collected after centrifuging at 13,300 g for 30 minutes at 4 ℃. Then, the protein concentrations of supernatant were measured by a spectrophotometer (NanoDrop 2000, Thermo, USA). About 30 µg total protein was separated by 10% SDS-PAGE. All proteins were transferred to a polyvinylidene fluoride membrane (IPVH00010, Millipore, USA). The membranes were incubated with 5% skim milk (D8340, Solarbio, China) for 2 hours at room temperature (RT) to block nonspecific antigens, then, incubated with BMAL1 (ARNTL) Rabbit Monoclonal Antibody (1:1000) or β-Actin Rabbit Monoclonal Antibody (1:2000, AF5003) at 4 ℃ overnight, followed by incubating with HRP-labeled Goat Anti-Rabbit IgG (1:1000, A0208) at RT for 1 hour. All anti-bodies were purchased from Beyotime (Shanghai, China). Finally, the protein bands were visualized by chemiluminescence and analyzed by ImageJ software (Version, 1.8.0, Softnic, USA).

### 2.5. Cell counting kit-8 (CCK-8) assay

The proliferation was analyzed by CCK-8. The transfected cells were seeded into 96-well plates at a concentration of 5000 cells per well and incubated in a 5% CO_2_ incubator at 37 ℃ for 24 hours. The 10 µL of CCK-8 solution per well (C0037, Beyotime, China) was added to transfected cells. Then, the cells were incubated in a 5% CO_2_ incubator at 37 ℃ for 1 hour. Finally, the optical density (OD) was measured at the wavelength of 450 nm on spectrophotometer (SpectraMax® M5, Molecular Devices, USA) to analyze proliferation of cells.

### 2.6. Wound healing

The invasion of cells was analyzed by wound healing. The transfected cells were seeded into 24-well plates at 1 × 10^4^/well. When the cells reached to 100% confluence, a wound was made uniformly from top to bottom on the well plate using the tip. The exfoliated cells were washed off with phosphate buffer solution. It was defined as 0 hours when a scratch was formed. Then, the cells were incubated in a 5% CO_2_ incubator at 37 ℃. Based on the growth of cells, wound healing after 36 hours of scratch formation was selected to assess cell migration. The area of scratch was visualized at 0 and 36 hours, and analyzed using ImageJ software. The migration rate was calculated using the following formula: migration rate (%) = (scratch area at 0 hours-scratch area at 36 hours)/scratch area at 0 hours × 100%.

### 2.7. Statistical analysis

All data was from 3 independent experiments and presented as the mean ± standard deviation. Student *t* test was performed to analyze the difference between 2 groups. The Tukey multiple comparisons of one-way ANOVA were executed to analyze the differences among groups. The Kaplan–Meier plotter was used to analyze the overall survival of liver cancer patients. The Spearman correlation analysis was used to analyze the relationship between ARNTL and ATG9B-4 expression. All data was analyzed using SPSS23.0 and GraphPad Prism 8.0 softwares. It was considered statistically significant when the *P*-value was <.05.

## 3. Results

### 3.1. ARNTL was identified in HepG2 cells with ATG9B-4

The HepG2 cells were transfected with or without pcDNA3.1-ATG9B-4, then the expression of ATG9B-4 was analyzed by RT-qPCR. As shown in Figure S1A, Supplemental Digital Content, https://links.lww.com/MD/O712, the expression of ATG9B-4 in the transfected pcDNA3.1-ATG9B-4 cells was significantly up-regulated compared with that in the transfected pcDNA3.1 cells (*P* < .0001), which suggested ATG9B-4 was successfully over-expressed in the HepG2 cells.

**Figure 1.**
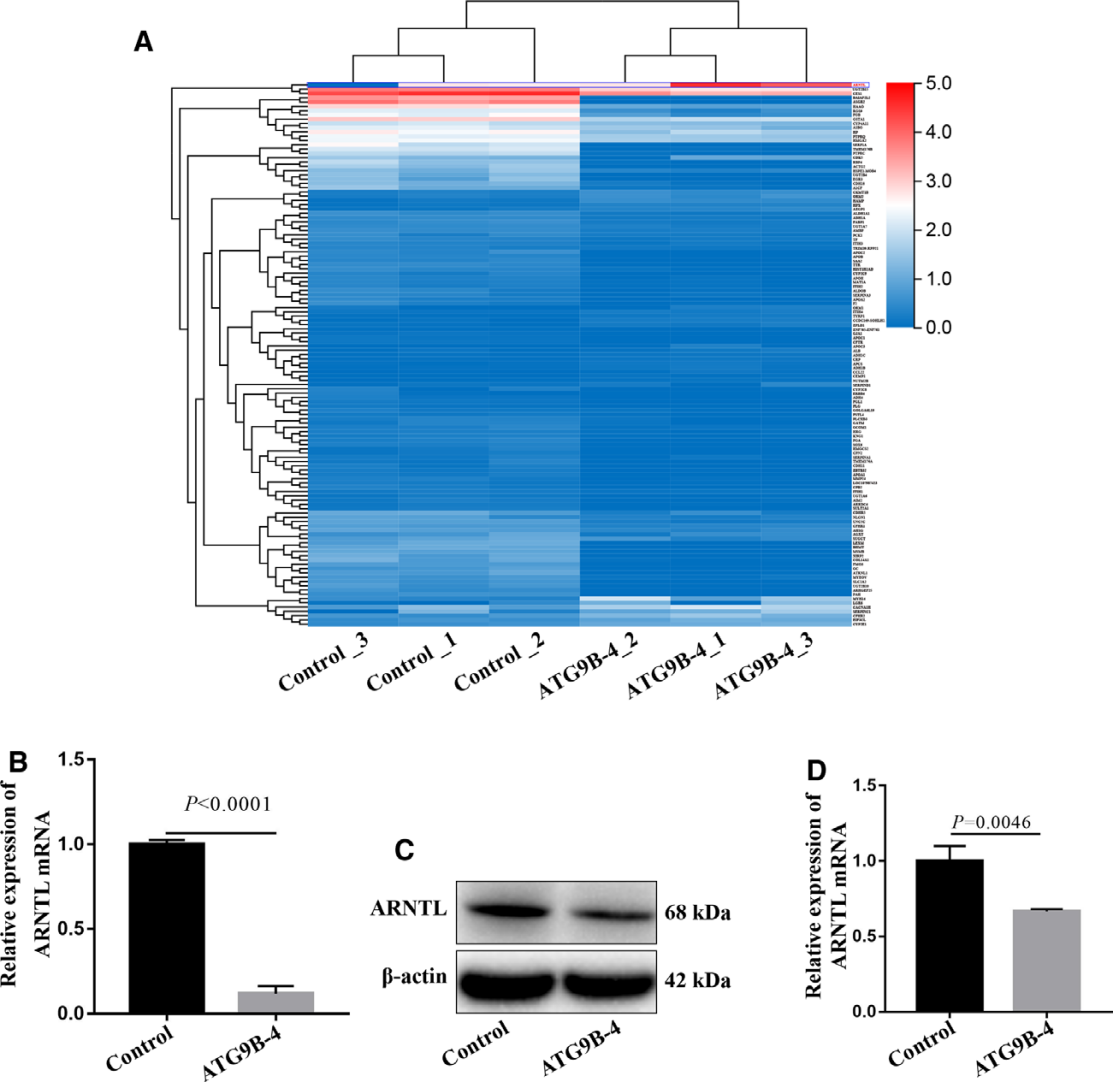
The expression of ARNTL was decreased in the HepG2 cells over-expressed ATG9B-4. The HepG2 were trasnfected with pcDNA3.1 (Control) or pcDNA3.1-ATG9B-4 (ATG9B-4). (A) Differential genes were identified by mRNA sequencing in the HepG2 cells. ARNTL was marked by red font and blue box. (B) ARNTL was one of the differentials expressed genes identified by sequencing, which suggested that the expression of ARNTL mRNA in the ATG9B-4 cells was significantly downregulated compared to that in the control cells (*t* test, *P* < .0001). (C and D) The expression of ARNTL was measured by western blotting in the cells with ATG9B-4, which showed that the expression of ARNTL protein in the ATG9B-4 cells was significantly decreased compared to that in the control cells (*t* test, *P* = .0046).

To analyze the target genes regulated by ATG9B-4, the genes expressions were sequenced in the ATG9B-4 HepG2 cells and control HepG2 cells. The 127 differential genes expression were identified in the ATG9B-4 cells (Fig. 1A and Table S1, Supplemental Digital Content, https://links.lww.com/MD/O713). Among them, ARNTL expression was down-regulated in the cells with ATG9B-4 (*P* < .0001) (Fig. 1B). In order to further confirm the differential expression of ARNTL in the cells, the western blot was performed. The ARNTL expression of cells with ATG9B-4 was decreased compared with that of control cells (*P* = .0046) (Fig. 1C and D). These results suggested that ATG9B-4 maybe regulate ARNTL expression in the HepG2 cells.

### 3.2. ARNTL is negatively correlated with ATG9B-4 expression and low expression of ARNTL indicates poor overall survival in liver cancer patients

**Figure 2.**
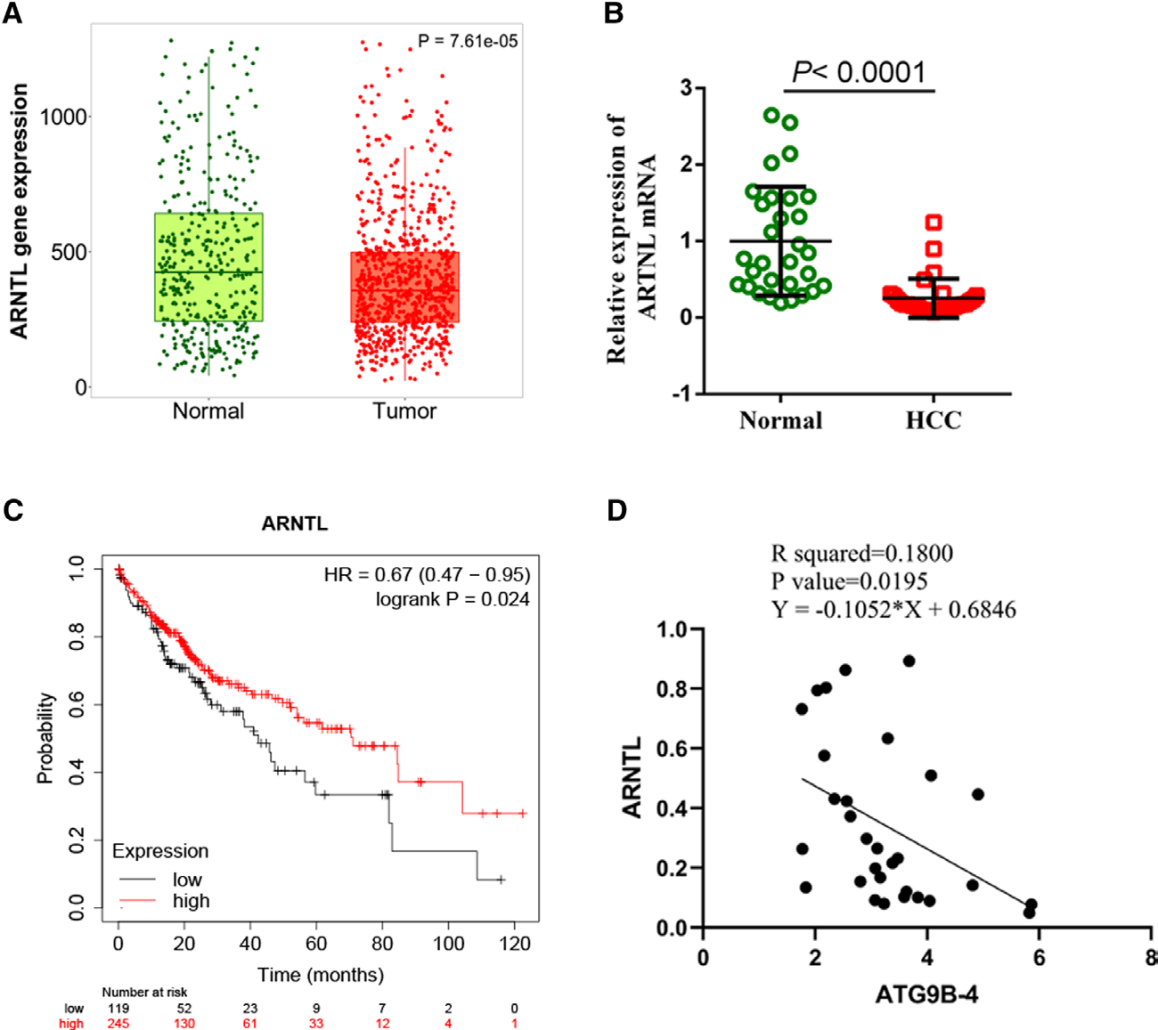
The expression of ARNTL was down-regulated in the patients with liver cancer. (A) The ARNTL gene expression was analyzed on the database (https://tnmplot.com), which indicated that the expression of ARNTL was markedly decreased in the tumor from the patients with liver cancer (*t* test, *P* = 7.61e-05). (B) The expression of ARNTL was measured by RT-qPCR in thirty patients with liver cancer, which suggested that the expression of ARNTL mRNA was dramatically decreased in the tumor from the patients with liver cancer (*t* test, *P* < .0001). (C) The overall survival of liver cancer was analyzed by Kaplan–Meier plotter assay. The result showed that the overall survival of patients with low ARNTL expression was significantly lower than that of patients with high ARNTL expression (K-M plotter, *P* = .024). (D) The correlation analysis was performed to clarify the relationship between ARNTL expression and ATG9B-4 expression in the patients with liver cancer, which revealed that the expression of ARNTL was negatively correlated with the expression of ATG9B-4 (Spearman, *P* = .0195).

To explore the expression of ARNTL in the patients with liver cancer, the data on ARNTL expression were collected from the database (https://tnmplot.com). The result showed that the expression of ARNTL was down-regulated in the liver cancer compared with that in the normal tissues (*P* = 7.61e-05) (Fig. 2A). Meanwhile, the expression of ARNTL was detected in the 30 liver cancer tissues and 30 normal tissues. Our results were consistent with the results of the database, which suggested that ARNTL was lowly expressed in the liver cancer patients (*P* < .0001) (Fig. 2B). To evaluate the effect of ARNTL expression on overall survival of liver cancer, the overall survival was analyzed by Kaplan–Meier plotter. The survival probability of liver cancer patients with high ARNTL expression was significantly higher than that of liver cancer patients with low ARNTL expression (*P* = .024), which revealed that low expression of ARNTL indicated poor overall survival of liver cancer patients (Fig. 2C). Our previous studies had shown that ATG9B-4 was highly expressed in patients with liver cancer.^[10]^ In order to analyze the relationship between ARNTL expression and ATG9B-4 expression in the liver cancer patients, the correlation analysis was performed. The expression of ARNTL was negatively correlated with the expression of ATG9B-4 in the patients with liver cancer (*P* = .0195) (Fig. 2D), while there was no significant correlation between ARNTL and ATG9B-4 in normal tissues from patients (*P* = .3580) (Figure S2, Supplemental Digital Content, https://links.lww.com/MD/O712), which manifested that ARNTL was negatively regulated by ATG9B-4 in the patients with liver cancer.

### 3.3. ATG9B-4 promoted proliferation and migration by down-regulating ARNTL in HepG2 cells

It was demonstrated that ATG9B-4 could promote the malignant characteristics of liver cancer cells.^[10]^ The HepG2 cells were transfected with pcDNA3.1-ARNTL. The expression of ARNTL in the pcDNA3.1-ARNTL cells was increased compared with that in the pcDNA3.1 cells (*P* = .0001), which indicated ARNTL was successfully over-expressed in the HepG2 cells (Figure S1B, Supplemental Digital Content, https://links.lww.com/MD/O712).

**Figure 3.**
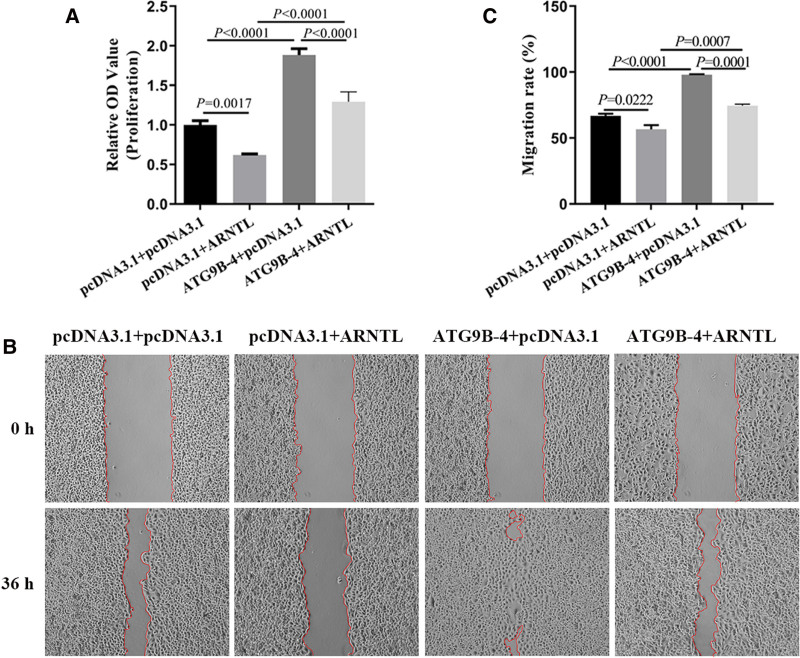
ARNTL could inhibit proliferation and migration induced by ATG9B-4 in liver cancer cells. The HepG2 cells were transfected with ATG9B-4 and/or ARNTL. (A) The proliferation was analyzed by CCK-8 assay. The result showed that ARNTL significantly decreased the increase of proliferation induced by ATG9B-4 in liver cancer cells (one-way ANOVA, *P* < .0001). (B and C) The migration was analyzed by wound healing assay, which suggested that ARNTL significantly inhibited the increase of migration induced by ATG9B-4 in liver cancer cells (one-way ANOVA, *P* = .0001).

To verify whether ATG9B-4 promoted the proliferation and invasion of liver cancer cells by regulating ARNTL expression, the HepG2 cells were co-transfected ATG9B-4 with or without ARNTL. The OD value of cells was determined 24 hours after transfection. The OD value of cells transfected with ARNTL was significantly lower than that of control cells (*P* = .0017), and the OD value of cells transfected with ATG9B-4 was markedly higher than that of control cells (*P* < .0001), however, the OD value of cells transfected with ATG9B-4 and ARNTL were significantly weakened compared with cells transfected ATG9B-4 (*P* < .0001), which suggested that ARNTL could weaken the cell proliferation induced by ATG9B-4 (Fig. 3A). The migration of cells was wound healing. The wound area of cells transfected with ARNTL was wider than that of cells with control cells (*P* = .0222), and the wound area of cells transfected with ATG9B-4 was obviously narrower than that of control cells (*P* < .0001), however, the wound area of cells transfected with ATG9B-4 and ARNTL were remarkably wider than that of cells transfected with ATG9B-4 (*P* = .0001) (Fig. 3B), which indicated that ARNTL could inhibit the promoting of ATG9B-4 on cell migration in liver cancer cells (Fig. 3C)

### 3.4. ARNTL down-regulated the expression of CDK5 in liver cancer cells

Our previous study had confirmed that ATG9B-4 could promote the malignant characteristics of liver cancer cells by up-regulating the expression of CDK5.^[10]^ And the above study also concluded that ATG9B-4 accelerated the proliferation and migration by down-regulating the expression of ARNTL. To explore whether ARNTL can regulate the CDK5 expression, ARNTL and/or CDK5 were transfected into HepG2 cells.

**Figure 4.**
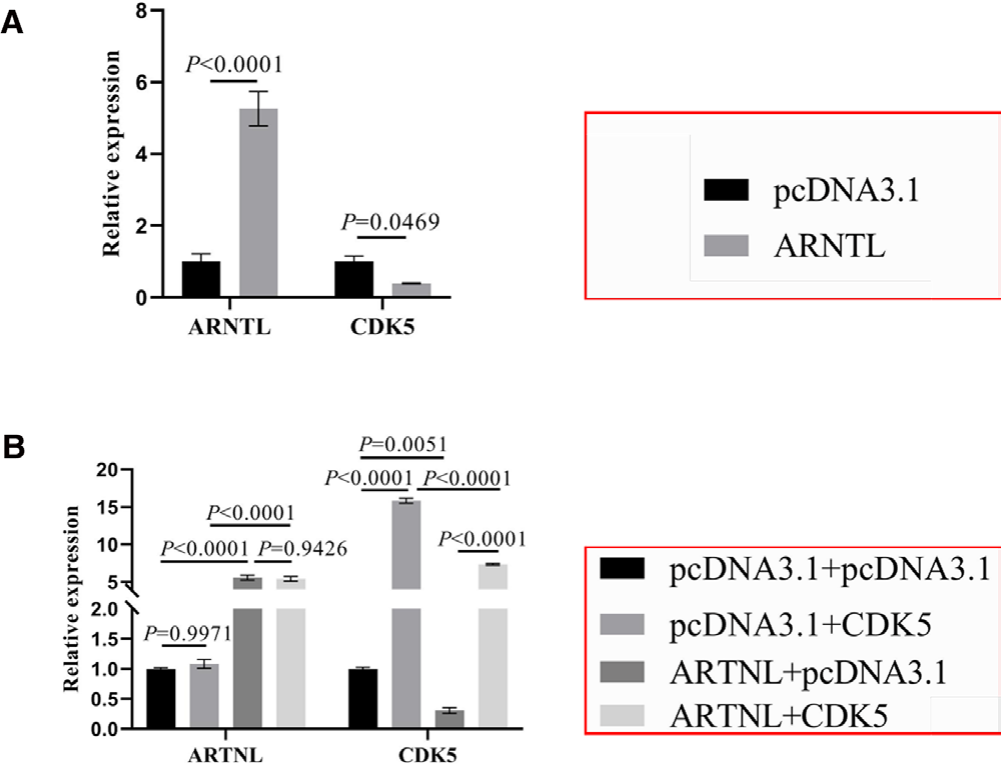
ARNTL inhibited the expression of CDK5 in the liver cancer cells. The expression of ARNTL and CDK5 were analyzed by RT-qPCR in the HepG2 cells transfected with or without ARNTL and cells transfected with or without ARNTL and CDK5. (A) Compared with pcDNA3.1 cells, the expression of ARNTL mRNA was significantly increased (*t* test, *P* < .0001), but the expression of CDK5 was significantly deceased in the cells transfected with ARNTL (*t* test, *P* = .0469). (B) The expression of ARNTL mRNA was no significant difference between pcDNA3.1 cells and CDK5 cells (one-way ANOVA, *P* = .9971), ARNTL + pcDNA3.1 cells and ARNTL + CDK5 cells (one-way ANOVA, *P* = .9426). The expression of CDK5 in the pcDNA3.1 + CDK5 cells was up-regulated compared with that in the pcDNA3.1 cells (one-way ANOVA, *P* < .0001). The expression of CDK5 in the ARNTL + CDK5 cells was significantly decreased compared with that in the pcDNA3.1 + CDK5 cells (one-way ANOVA, *P* < .0001).

In the cells transfected with ARNTL, the expression of ARNTL was up-regulated and that of CDK5 was down-regulated (respectively, *P* < .0001 and *P* = .0469) (Fig. 4A). Then, HepG2 cells were transfected with or without ARNTL and CDK5. The expressions of ARNTL and CDK5 were analyzed by RT-qPCR. In terms of ARNTL expression, the ARNTL expression of cells transfected with CDK5 was same as that of control cells (*P* = .9971) and the expression of ARNTL in cells transfected with ARNTL and CDK5 did not differ from that of cells transfected with ARNTL (*P* = .9426), which suggested that CDK5 could not regulate the ARNTL expression (Fig. 4B). In terms of CDK5 expression, compared with control cells, the expression of CDK5 was remarkably increased in the cells transfected with CDK5 (*P* < .0001, Fig. 4B and Figure S1C, Supplemental Digital Content, https://links.lww.com/MD/O712) and that was decreased in the cells transfected with ARNTL (*P* = .0051, Fig. 4B). The CDK5 expression of cells transfected with ARNTL and CDK5 was up-regulated compared to that of cells transfected with ARNTL (*P* < .0001), and was lower than that of cells transfected with CDK5 (*P* < .0001), which indicated that CDK5 could partly inhibit the down-regulation of CDK5 induced by ARNTL (Fig. 4B). These results have shown that ARNTL down-regulated the expression of CDK5 in liver cancer cells.

### 3.5. Down-regulation of CDK5 attenuated promotion of proliferation and migration induced by ATG9B-4 though ARNTL–CDK5 axis in HepG2 cells

**Figure 5.**
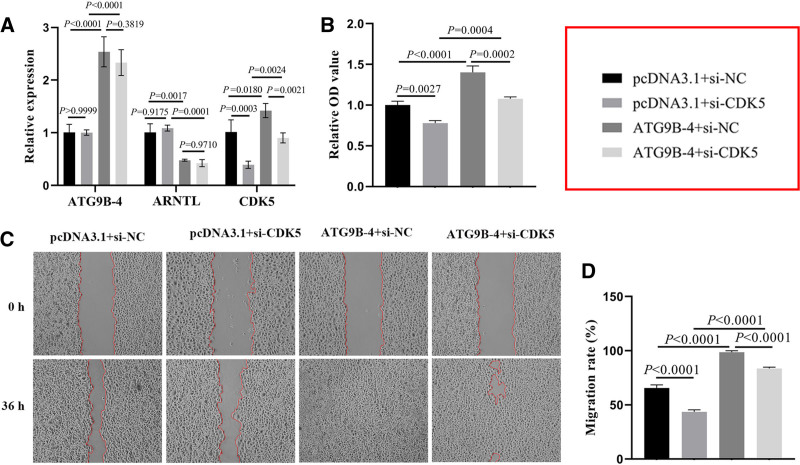
CDK5 knockdown attenuates the promotion of proliferation and migration induced by ATG9B-4 in liver cancer cells. The HepG2 cells were transfected with si-CDK5 and/or ATG9B-4. (**A**) The expression of ATG9B-4, ARNTL and CDK5 were detected by RT-qPCR. The result showed that ATG9B-4 could downregulate the expression of ARNTL (one-way ANOVA, *P* = .0017) and upregulate the expression of CDK5 (one-way ANOVA, *P* = .018), meanwhile loss of CDK5 was not involved in the regulation of ATG9B-4 (one-way ANOVA, *P* = .3819) and ARNTL (one-way ANOVA, *P* = .9710) expression, but could inhibit the up-regulation of CDK5 (one-way ANOVA, *P* = .0021) induced by ATG9B-4. (B) The OD value was analyzed by CCK-8assay to evaluate proliferation of cells, which showed that down-regulation of CDK5 could inhibit the proliferation (one-way ANOVA, *P* = .0027) and reduce increase of proliferation induced by ATG9B-4 (one-way ANOVA, *P* = .0002) in liver cancer cells. (C and D) The migration was analyzed by wound healing assay.The results showed that down-regulation of CDK5 could inhibit the migration (one-way ANOVA, *P* < .0001) and reduce increase of migration induced by ATG9B-4 (one-way ANOVA, *P* < .0001) in liver cancer cells.

To determine whether si-CDK5 interferes with CDK5 expression, the HepG2 cells were transected with si-control (si-NC) or si-CDK5. The expression of CDK5 was analyzed by RT-qPCR assay. Compared with si-NC cells, the CDK5 expression of the si-CDK5 cells was markedly down-regulated (*P* = .0023, Figure S1D, Supplemental Digital Content, https://links.lww.com/MD/O712). To investigate the role of ATG9B-4 in promoting malignant features of liver cancer cells through regulating ARNTL–CDK5 axis, the HepG2 cells were transfected with or without ATG9B-4 and si-CDK5. The expression of ATG9B-4, ARNTL, and CDK5 were detected by RT-qPCR assay. The expression of ATG9B-4, ARNTL, and CDK5 were shown in Fig. 5A. Compared with control cells, the expression of ARNTL was down-regulated (*P* = .0017), meanwhile, the expression of ATG9B-4 and CDK5 was significantly up-regulated in the cells transfected with ATG9B-4 (respectively, *P* < .0001 and *P* = .0180); the expression of CDK5 was down-regulated (*P* = .0003), however, there was no significant difference in the expression of ATG9B-4 and ARNTL in cells transfected with si-CDK5 (respectively, *P* > .9999 and *P* = .9175). As expected, the expression of CDK5 was down-regulated in the cells transfected with si-CDK5 (*P* = .0003), and that was up-regulated in the cells transfected with ATG9B-4 (*P* = .0180), however, the CDK5 expression of cells transfected with ATG9B-4 and si-CDK5 was higher than that of cells transfected with si-CDK5 (*P* = .0024), and lower than that of cells transfected with ATG9B-4 (*P* = .0021), which suggested that down-regulation of CDK5 was not involved in the regulation of ATG9B-4 and ARNTL expression, but could inhibit the up-regulation of CDK5 induced by ATG9B-4.

The proliferation was analyzed by CCK-8 assay. The proliferation was shown in Figure 5B. Compared with control cells, the OD value in the cells transfected with si-CDK5 was significantly decreased (*P* = .0027), and the OD value in the cells transfected with ATG9B-4 was markedly increased (*P* < .0001). The OD value in the cells transfected with ATG9B-4 and si-CDK5 was higher than that in the cells transfected with si-CDK5 (*P* = .0004), and it was lower than that in the cells transfected with ATG9B-4 (*P* = .0002). The results of OD value indicated that CDK5 knockdown effectively suppressed the proliferation of liver cancer cells induced by ATG9B-4.

The migration was analyzed by wound healing. As shown in Figure 5C, the area of wound in the cells transfected with ATG9B-4 was completely healed, and the area of wound in the cells transfected with si-CDK5 was wider than that in control cells (*P* < .0001). In the cells transfected with ATG9B-4 and si-CDK5, the area of wound was narrower than that in the si-CDK5 cells (*P* < .0001), and it was wider than that in ATG9B-4 cells (*P* < .0001), which suggested that CDK5 knockdown partially attenuated promotion of migration induced by ATG9B-4 in liver cancer cells (Fig. 5D).

## 4. Discussion

Liver cancer is a common malignant tumor of the digestive system that accounts for 75% to 85% of liver malignancies.^[3]^ The survival rate of liver cancer patients has significantly improved due to advancements in surgical, chemotherapy and radiotherapy treatment techniques and the widespread use of immunotherapy. Recent research has shown that the combination of tyrosine kinase inhibitors and immune checkpoint inhibitors increases antitumor efficacy and prolongs overall survival in patients with advanced liver cancer by targeting multiple genes, such as PD-1, PD-L1, CTLA-4, LAG-3, and TIM3.^[12]^ However, due to the complex pathogenesis and limited treatment strategies available for liver cancer, the overall survival rate of liver cancer patients is unsatisfactory.^[4]^ Therefore, it is very necessary to conduct in-depth research on the pathogenesis of liver cancer in an attempt to identify new biomarkers or new regulatory molecular mechanisms.

Increasing evidence has shown that lncRNAs such as lnc ZEB2-19,^[9]^ lnc TSPAN12,^[13]^ lnc CCNH-8,^[14]^ and linc01116,^[15]^ are abnormally expressed in liver cancer and commonly used as biomarker for the diagnosis and prognosis of liver cancer, such as CASC9,^[16]^ FTO-IT1,^[17]^ SOCS2-AS1.^[18]^ Among these lncRNAs, ATG9B-4 was highly expressed in liver cancer and promoted the growth, TNM stage and metastasis of liver cancer in our previous study.^[10]^ However, the mechanism by which ATG9B-4 regulates malignant characteristics of liver cancer still needs to be further studied.

CDK5 encodes a proline-directed serine/threonine kinase that is a member of the CDK family of proteins and is ubiquitously expressed in tissues throughout the body and regulates the cell cycle.^[19]^ CDK5 has been shown to act as an oncogene or anti-oncogene.^[20]^ Several studies have shown that CDK5 is abnormally expressed in liver cancer and promotes or inhibits liver cancer progression.^[20–22]^ Our previous study showed that CDK5 is up-regulated by ATG9B-4 and promotes the proliferation and migration of liver cancer cells.^[10]^ Based on the negative regulation of target genes by lncRNA, CDK5 is not directly regulated but rather indirectly regulated by ATG9B-4 in liver cancer cells. To explore the specific molecular mechanism by which ATG9B-4 regulates CDK5, we performed mRNA sequencing of liver cancer cells overexpressing ATG9B-4. In this study, ARNTL was identified as being down-regulated in liver cancer cells overexpressing ATG9B-4.

ARNTL is a well-known gene that regulates the circadian rhythm.^[23,24]^ Abnormal expression of ARNTL can promote the tumorigenesis, which is also widely recognized. ARNTL expression was downregulated in nasopharyngeal carcinoma cells and tissues due to the hypermethylation of ARNTL, and the overexpression of ARNTL inhibited the proliferation of nasopharyngeal carcinoma cells both in vitro and in vivo.^[25]^ ARNTL expression is reduced in non-small cell lung cancer, and it was confirmed that ARNTL initiates the transcription of GUCY1A2 to attenuate the progression of non-small cell lung cancer through miR-200c-3p/PTEN signaling.^[26]^ ARNTL activated autophagy in an AKT/mTOR pathway-dependent manner, thereby decreasing cell proliferation, enhancing cell death and hindering the migration of oral cancer cells.^[27,28]^ In this study, the expression of ARNTL was reduced in both liver cancer patients and ATG9B-4-overexpressing liver cancer cells. Patients with low ARNTL expression had poor overall survival. Moreover, ARNTL expression was negatively correlated with ATG9B-4 expression in liver cancer patients, and ARNTL suppressed the promoting effect of ATG9B-4 on the proliferation and migration of liver cancer cells. Our previous studies showed that ATG9B-4 indirectly up-regulated the expression of CDK5,^[10]^ and this study showed that ATG9B-4 could downregulate ARNTL expression in liver cancer cells. The regulatory relationship between ARNTL and CDK5 still needs further study. Our results suggested that the expression of CDK5 decreased in ARNTL-overexpressing liver cancer cells but that the overexpression of CDK5 did not affect ARNTL expression. These results indicated that ARNTL could regulate the expression of CDK5, which was consistent with the results of previous studies.^[25]^ However, the mechanism by which ARNTL regulates the expression of CDK5 remains to be further explored.

In this study, ARNTL expression was down-regulated in liver cancer patients and in cells overexpressing ATG9B-4. Liver cancer patients with low ARNTL expression had poor overall survival. Moreover, ARNTL attenuated the ATG9B-4-induced increase in cell proliferation and migration in liver cancer cells. ARNTL negatively regulatse CDK5 expression in liver cancer cells. Finally, loss of CDK5 abrogated the promotion of proliferation and migration by ATG9B-4 in liver cancer cells.

## 5. Conclusion

In conclusion, these results showed that ATG9B-4 deteriorated the proliferation and migration of liver cancer cells in an ARNTL–CDK5 pathway-dependent manner. There are some limitations in this study. Firstly, our study only confirmed that ATG9B-4 promoted the malignant characteristics of liver cancer by ARNTL–CDK5 pathway in vitro, which has not yet been testified in vivo. Secondly, this study showed that ATG9B-4 promoted the proliferation and migration of liver cancer cells by ARNTL–CDK5 pathway, but whether ATG9B-4 directly regulated ARNTL–CDK5 pathway remains to be further explored. Thirdly, the signaling pathway regulated by lncRNAs, such as lncRNA PVT1/miR-214-3p/GPX4, are expected to become potential targets for the treatment of liver cancer.^[29]^ However, there are still no effective therapies by targeting ATG9B-4/ARNTL/CDK5. So, our further research focuses on identifying effective therapies that target the ATG9B-4/ARNTL/CDK5 signaling pathway.

## Acknowledgments

We thank all the listed authors in the manuscript.

## Author contributions

**Conceptualization:** Ming Li, Jiefu Tang.

**Data curation:** Ming Li, Wenxiong Zhu, Pinyue Liu.

**Funding acquisition:** Ming Li, Jiefu Tang, Wenxiong Zhu.

**Formal analysis:** Changshen Cheng.

**Investigation:** Ming Li, Lili Guo.

**Methodology:** Lili Guo.

**Project administration:** Zhongcheng Mo.

**Software:** Pinyue Liu.

**Supervision:** Zhongcheng Mo.

**Validation:** Ming Li.

**Visualization:** Changshen Cheng.

**Writing – original draft:** Ming Li, Jiefu Tang, Wenxiong Zhu.

**Writing – review & editing:** Ming Li, Zhongcheng Mo.

## Supplementary Material


