# Supplementary material for: ATG9B-4 accelerates the proliferation and migration of liver cancer cells in an ARNTL–CDK5 pathway-dependent manner: A case–control study
Source: Medicine (Baltimore). 2025 Apr 18;104(16):e42227. doi: 10.1097/MD.0000000000042227 (PMC12014037; doi:10.1097/MD.0000000000042227)

**Suppl. Figure 1. The expression of ATG9B-4, ARNTL and CDK5 were analyzed by RT-qPCR in** **liver cancer cells.** The HepG2 cells were transfected with overexpression plasmids (ATG9B-4, ARNTL and CDK5) or si-CDK5. Then the expression of target genes were analyzed by RT-qPCR. Compared to cells transfected with pcDNA3.1, the expression of ATG9B-4 (**A**), ARNTL (**B**) and CDK5 (**C**) were respectively upregulated in the cells transfected with pcDNA3.1-ATG9B-4, pcDNA3.1-ARNTL and pcDNA3.1-CDK5 (Respectively, *t*-test, *P*<0.0001, *P*=0.0001, *P*<0.0001). (**D**) The expression of CDK5 in the si-CDK5 cells was downregulated compared with si-NC cells (*t*-test, *P=*0.0023).


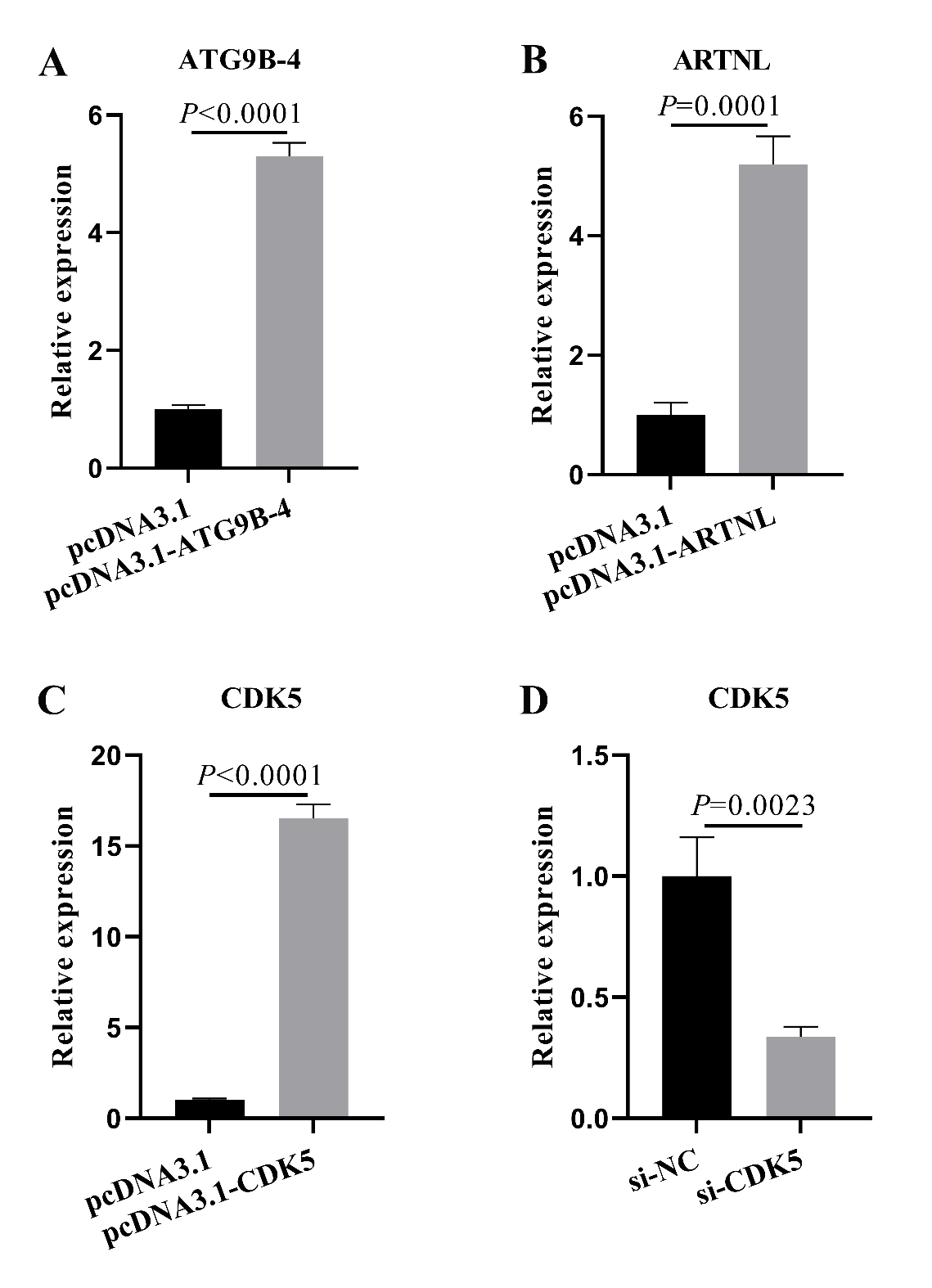


**Suppl. Figure 2. The correlation analysis was performed to clarify the relationship between ARNTL expression and ATG9B-4 expression in normal tissues from patients with liver cancer.** The expression of ARNTL was no significantly correlated with the expression of ATG9B-4 in normal tissues from patients (Spearman, *P*=0.3580)


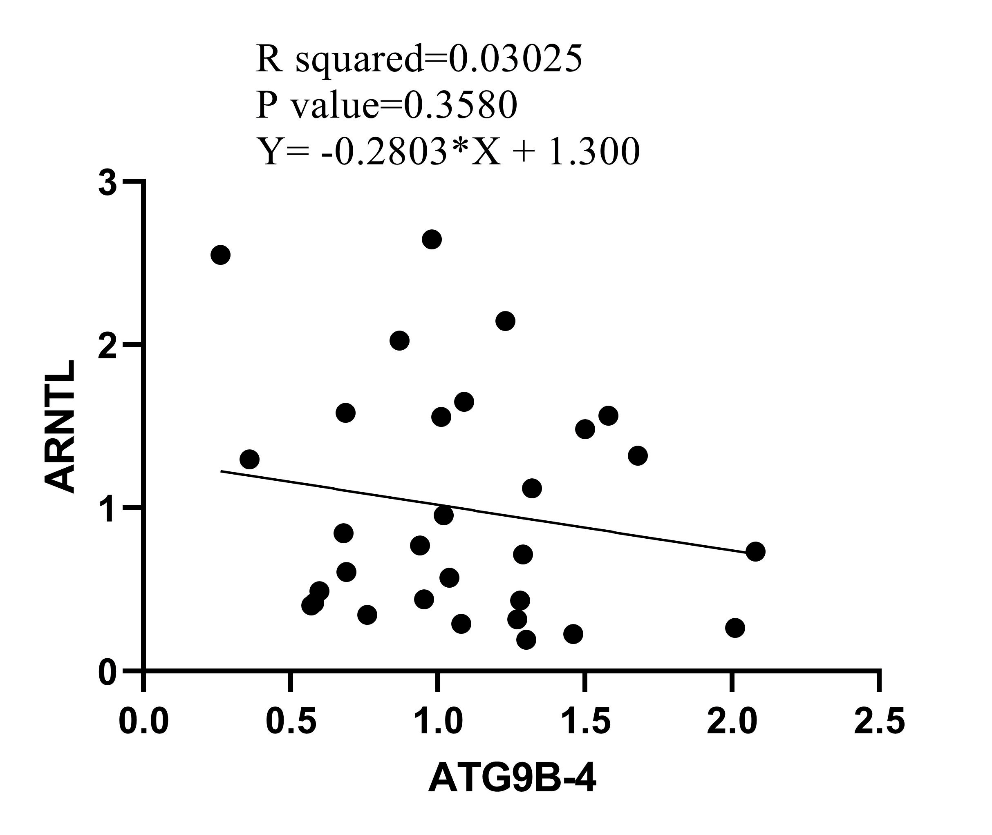

Supplement: Supplementary file 1 [file medi-104-e42227-s001.docx]
