# Supplementary material for: ATG9B-4 accelerates the proliferation and migration of liver cancer cells in an ARNTL–CDK5 pathway-dependent manner: A case–control study
Source: Medicine (Baltimore). 2025 Apr 18;104(16):e42227. doi: 10.1097/MD.0000000000042227 (PMC12014037; doi:10.1097/MD.0000000000042227)
Supplement: Supplementary file 2 [file medi-104-e42227-s002.doc]

**Suppl. Table 1 The 127 differential genes expression were identified by mRNA Sequencing in the HepG2 cells transfected with ATG9B-4**

| **Gene Symbol** | **Type** | **control_1** | **control_2** | **control_3** | **ATG9B-4_1** | **ATG9B-4_2** | **ATG9B-4_3** |
| --- | --- | --- | --- | --- | --- | --- | --- |
| ARNTL | mRNA | 3.4 | 3.48 | 3.58 | 0.23 | 0.48 | 0.52 |
| UGT2B15 | mRNA | 0.1 | 0.14 | 0.14 | 0 | 0.04 | 0 |
| CES1 | mRNA | 0.12 | 0.18 | 0.06 | 0 | 0 | 0 |
| BAIAP2L2 | mRNA | 0.07 | 0.15 | 0.06 | 0.3 | 0.27 | 0.38 |
| ASGR2 | mRNA | 0.12 | 0.2 | 0.17 | 0 | 0 | 0 |
| HAAO | mRNA | 0.03 | 0 | 0.25 | 0 | 0 | 0 |
| RGS9 | mRNA | 3.22 | 2.56 | 2.82 | 1.52 | 1.58 | 1.34 |
| FGB | mRNA | 0.8 | 0.77 | 0.88 | 0.12 | 0.2 | 0.17 |
| GSTA1 | mRNA | 0.28 | 0.37 | 0.31 | 0 | 0 | 0 |
| CYP4A11 | mRNA | 0.21 | 0.23 | 0.15 | 0 | 0.02 | 0.02 |
| A1BG | mRNA | 0.37 | 0.44 | 0.5 | 0.1 | 0.13 | 0.13 |
| HP | mRNA | 2.99 | 3.33 | 3.27 | 0.04 | 0 | 0 |
| PTPRQ | mRNA | 0.04 | 0.04 | 0.02 | 0.15 | 0.1 | 0.06 |
| HMGA2 | mRNA | 1.04 | 0.7 | 1 | 0.45 | 0.18 | 0.15 |
| SERF1A | mRNA | 5.71 | 5.42 | 0 | 22.22 | 5.74 | 16.29 |
| TMEM176B | mRNA | 0.23 | 0.3 | 0.18 | 0 | 0 | 0 |
| PTPRC | mRNA | 0.08 | 0.08 | 0.1 | 0.14 | 0.18 | 0.19 |
| CDK5 | mRNA | 0.11 | 0.09 | 0.05 | 0.25 | 0.26 | 0.26 |
| RBP4 | mRNA | 4.29 | 5.13 | 5.54 | 2.55 | 1.57 | 2.03 |
| ACTG2 | mRNA | 3.79 | 3.04 | 3.67 | 1.77 | 1.95 | 1.16 |
| HSPE1-MOB4 | mRNA | 0 | 0 | 0.16 | 0.22 | 0 | 0.23 |
| UGT2B4 | mRNA | 0.16 | 0.15 | 0.09 | 0.02 | 0 | 0 |
| EGR3 | mRNA | 0.27 | 0.18 | 0.3 | 0.11 | 0.13 | 0.08 |
| CDH18 | mRNA | 0.03 | 0.02 | 0.04 | 0.15 | 0.11 | 0.15 |
| A1CF | mRNA | 0.03 | 0.03 | 0.03 | 0 | 0 | 0.01 |
| CKMT1B | mRNA | 0.59 | 0.97 | 0.48 | 0.46 | 0.13 | 0.43 |
| ORM2 | mRNA | 0.55 | 0.52 | 0.45 | 0 | 0 | 0 |
| HAMP | mRNA | 1.07 | 0.95 | 0.9 | 0 | 0 | 0 |
| HPX | mRNA | 0.49 | 0.65 | 0.63 | 0.03 | 0.06 | 0.11 |
| AZGP1 | mRNA | 0.23 | 0.15 | 0.49 | 0.04 | 0 | 0 |
| ALDH1A1 | mRNA | 0.23 | 0.26 | 0.33 | 0.06 | 0 | 0 |
| ADH1A | mRNA | 0.41 | 0.34 | 0.41 | 0 | 0 | 0 |
| FABP1 | mRNA | 0.65 | 0.99 | 1.12 | 0 | 0 | 0 |
| UGT1A7 | mRNA | 4.26 | 4.25 | 4.07 | 1.96 | 2.13 | 1.95 |
| AMBP | mRNA | 1.15 | 1.34 | 1.58 | 0.06 | 0.17 | 0 |
| PCK1 | mRNA | 0.2 | 0.33 | 0.2 | 0.08 | 0.04 | 0.05 |
| TF | mRNA | 0.67 | 1.02 | 1.26 | 0 | 0.04 | 0.02 |
| ITIH3 | mRNA | 0.08 | 0.3 | 0.13 | 0 | 0 | 0 |
| TRIM39-RPP21 | mRNA | 1.29 | 0.25 | 0 | 1.17 | 0.91 | 2.02 |
| APOC2 | mRNA | 1.46 | 1.96 | 2.16 | 0 | 0 | 0 |
| APOB | mRNA | 0.07 | 0.07 | 0.11 | 0 | 0 | 0 |
| SAA2 | mRNA | 0.4 | 0.68 | 0.61 | 1.91 | 1.39 | 1.2 |
| TTR | mRNA | 1.59 | 1.69 | 2.64 | 0 | 0.05 | 0 |
| HIST1H2AD | mRNA | 1.6 | 0.74 | 0.69 | 3.19 | 2.18 | 2.37 |
| CYP2C9 | mRNA | 0.15 | 0.1 | 0.2 | 0.02 | 0 | 0 |
| APOH | mRNA | 0.96 | 1.47 | 1.63 | 0 | 0 | 0 |
| MAT1A | mRNA | 0.08 | 0.06 | 0.15 | 0.01 | 0 | 0 |
| ITIH2 | mRNA | 0.2 | 0.26 | 0.23 | 0 | 0 | 0 |
| ALDOB | mRNA | 0.64 | 0.99 | 0.72 | 0 | 0 | 0.02 |
| SERPINA3 | mRNA | 0.41 | 0.6 | 0.74 | 0 | 0 | 0 |
| APOA2 | mRNA | 8.97 | 9.58 | 10.87 | 0 | 0 | 0 |
| F2 | mRNA | 0.16 | 0.3 | 0.3 | 0 | 0 | 0 |
| ORM1 | mRNA | 0.75 | 1.72 | 1.56 | 0 | 0 | 0 |
| ITIH4 | mRNA | 0.31 | 0.28 | 0.43 | 0.15 | 0.1 | 0.14 |
| TYRP1 | mRNA | 0.27 | 0.2 | 0.17 | 0.35 | 0.5 | 0.58 |
| CCDC169-SOHLH2 | mRNA | 0 | 0 | 0 | 0.25 | 0 | 0 |
| ZPLD1 | mRNA | 0.05 | 0.01 | 0.07 | 0.12 | 0.15 | 0.22 |
| ZNF765-ZNF761 | mRNA | 0.19 | 0.16 | 0.13 | 0.07 | 0.06 | 0.07 |
| GJA1 | mRNA | 0.13 | 0.1 | 0.04 | 0.4 | 0.45 | 0.38 |
| APOC1 | mRNA | 3.53 | 4.85 | 4.28 | 0.36 | 0.76 | 0.45 |
| CFTR | mRNA | 0.01 | 0.04 | 0.04 | 0.14 | 0.11 | 0.08 |
| APOC3 | mRNA | 2.6 | 3.08 | 4.93 | 0 | 0 | 0 |
| ALB | mRNA | 10.51 | 12.6 | 13.93 | 0 | 0 | 0.02 |
| ADH1C | mRNA | 0.19 | 0.18 | 0.11 | 0 | 0 | 0 |
| CRP | mRNA | 0.3 | 0.29 | 0.34 | 0 | 0 | 0 |
| APCS | mRNA | 0.37 | 0.41 | 0.58 | 0 | 0 | 0 |
| ADH1B | mRNA | 0.44 | 0.58 | 0.64 | 0 | 0 | 0 |
| CCL22 | mRNA | 6.9 | 7.02 | 7.32 | 2.72 | 2.27 | 2.9 |
| CEMP1 | mRNA | 0 | 0.39 | 0.07 | 0 | 1.19 | 1.55 |
| NUTM2B | mRNA | 0.11 | 0.24 | 0.15 | 0.11 | 0 | 0.03 |
| SERPIND1 | mRNA | 0.21 | 0.24 | 0.33 | 0.06 | 0.04 | 0 |
| CYP2C8 | mRNA | 0.17 | 0.23 | 0.31 | 0.05 | 0.03 | 0 |
| ERBB4 | mRNA | 0.01 | 0.02 | 0 | 0.07 | 0.05 | 0.05 |
| ADH4 | mRNA | 0.32 | 0.31 | 0.44 | 0.02 | 0.07 | 0 |
| FGL1 | mRNA | 0.31 | 0.15 | 0.37 | 0.08 | 0 | 0.04 |
| PLG | mRNA | 0.07 | 0.21 | 0.21 | 0 | 0 | 0 |
| GOLGA6L19 | mRNA | 0.1 | 0.08 | 0.03 | 0 | 0.17 | 0.35 |
| FSTL4 | mRNA | 0.78 | 0.71 | 0.91 | 0.26 | 0.33 | 0.41 |
| PLCXD3 | mRNA | 0.58 | 0.46 | 0.39 | 1.16 | 0.83 | 1.01 |
| GATM | mRNA | 0.43 | 0.5 | 0.35 | 0.13 | 0.16 | 0.17 |
| GCOM1 | mRNA | 1.34 | 1.22 | 1.91 | 0.98 | 0 | 0.92 |
| HRG | mRNA | 0.81 | 0.89 | 0.96 | 0 | 0 | 0 |
| KNG1 | mRNA | 0.6 | 0.84 | 0.51 | 0 | 0 | 0 |
| FGA | mRNA | 1.13 | 1.82 | 1.51 | 0.24 | 0.3 | 0.36 |
| SOX6 | mRNA | 0.04 | 0.03 | 0.03 | 0.1 | 0.08 | 0.09 |
| HMGCS2 | mRNA | 0.2 | 0.19 | 0.39 | 0 | 0 | 0 |
| CPN2 | mRNA | 0.09 | 0.06 | 0.06 | 0 | 0 | 0 |
| SERPINA1 | mRNA | 6.23 | 5.47 | 6.5 | 0.82 | 1.02 | 0.86 |
| TMEM176A | mRNA | 0.28 | 0.5 | 0.37 | 0 | 0 | 0 |
| CDH11 | mRNA | 0.27 | 0.25 | 0.32 | 0.15 | 0.07 | 0.07 |
| ZBTB32 | mRNA | 12.06 | 12.41 | 13.15 | 5.67 | 6.71 | 5.73 |
| APOA1 | mRNA | 2.37 | 2.76 | 2.55 | 0 | 0.05 | 0 |
| MMP24 | mRNA | 0.48 | 0.55 | 0.39 | 1.08 | 1 | 1.23 |
| LOC107987423 | mRNA | 0.33 | 0.16 | 0.43 | 0.04 | 0 | 0 |
| CPB2 | mRNA | 0.11 | 0.18 | 0.18 | 0.03 | 0 | 0 |
| ITIH1 | mRNA | 0.21 | 0.21 | 0.2 | 0 | 0.04 | 0.05 |
| UGT1A6 | mRNA | 0 | 0.24 | 0.27 | 0 | 0 | 0.03 |
| AIM2 | mRNA | 0.87 | 1.05 | 0.71 | 0.24 | 0.63 | 0.41 |
| ARRDC4 | mRNA | 0.26 | 0.21 | 0.19 | 0.38 | 0.5 | 0.47 |
| SULT2A1 | mRNA | 0.25 | 0.14 | 0.23 | 0.05 | 0 | 0.04 |
| CDHR5 | mRNA | 0.12 | 0.11 | 0.23 | 0.02 | 0.07 | 0 |
| NLGN1 | mRNA | 0.01 | 0 | 0.01 | 0.03 | 0.06 | 0.05 |
| UNC5C | mRNA | 0.4 | 0.42 | 0.46 | 0.2 | 0.17 | 0.15 |
| CFHR1 | mRNA | 0.78 | 0.34 | 0.85 | 0.15 | 0.25 | 0.26 |
| AHSG | mRNA | 1.04 | 1.09 | 0.71 | 0 | 0 | 0 |
| AGXT | mRNA | 0.18 | 0.11 | 0.26 | 0 | 0 | 0 |
| SUGCT | mRNA | 0.06 | 0 | 0.02 | 0.18 | 0.17 | 0.19 |
| LEXM | mRNA | 0.22 | 0.16 | 0.1 | 0 | 0 | 0.04 |
| BHMT | mRNA | 0.11 | 0.09 | 0.16 | 0 | 0 | 0 |
| MSMB | mRNA | 22.37 | 23.34 | 18.51 | 8.33 | 11.42 | 8.91 |
| XIRP2 | mRNA | 0.02 | 0 | 0 | 0.12 | 0.02 | 0.05 |
| COL14A1 | mRNA | 0.05 | 0.03 | 0.02 | 0.09 | 0.08 | 0.09 |
| FMO3 | mRNA | 0.13 | 0.26 | 0.07 | 0 | 0 | 0 |
| GC | mRNA | 0.68 | 0.69 | 0.73 | 0 | 0 | 0 |
| ATRNL1 | mRNA | 0.08 | 0.01 | 0.01 | 0.11 | 0.04 | 0.17 |
| MYEOV | mRNA | 0.65 | 0.66 | 0.76 | 0.31 | 0.24 | 0.34 |
| SLC2A2 | mRNA | 0.1 | 0.08 | 0.11 | 0 | 0 | 0 |
| UGT2B10 | mRNA | 0.15 | 0.11 | 0.08 | 0 | 0 | 0.02 |
| ARHGEF25 | mRNA | 0.19 | 0.4 | 0.45 | 0.15 | 0.17 | 0.09 |
| PAH | mRNA | 0.08 | 0.08 | 0.23 | 0 | 0 | 0 |
| MYH14 | mRNA | 0.4 | 0.39 | 0.39 | 0.15 | 0.13 | 0.09 |
| LGR6 | mRNA | 3.62 | 3.78 | 4.12 | 1.91 | 2.04 | 1.78 |
| CACNA1H | mRNA | 0.38 | 0.41 | 0.39 | 0.17 | 0.24 | 0.16 |
| SERPINC1 | mRNA | 0.29 | 0.26 | 0.18 | 0 | 0.09 | 0.03 |
| CFHR2 | mRNA | 0.29 | 0.29 | 0.11 | 0 | 0.07 | 0 |
| EIF3CL | mRNA | 0.31 | 0.25 | 0.46 | 0.84 | 3.08 | 1.9 |
| CYP2E1 | mRNA | 1.07 | 1.06 | 1.85 | 0.08 | 0.11 | 0.03 |
